# Cysteinyl leukotriene receptor 1 facilitates tumorigenesis in a mouse model of colitis-associated colon cancer

**DOI:** 10.18632/oncotarget.16718

**Published:** 2017-03-30

**Authors:** Janina Osman, Sayeh Savari, Naveen Kumar Chandrashekar, Kishan Bellamkonda, Desiree Douglas, Anita Sjölander

**Affiliations:** ^1^ Division of Cell and Experimental Pathology, Department of Translational Medicine, Lund University, Skåne University Hospital, SE-205 02, Malmö, Sweden

**Keywords:** CysLT_1_ receptor, LTD_4_ signaling, colon cancer, colitis-associated colon cancer (CAC), inflammation

## Abstract

Cysteinyl leukotriene receptor 1 (CysLT_1_R) has been shown to be up-regulated in the adenocarcinomas of colorectal cancer patients, which is associated with a poor prognosis. In a spontaneous model of colon cancer, CysLT_1_R disruption was associated with a reduced tumor burden in double-mutant female mice (*Apc^Min/+/^Cysltr1^−/−^*) compared to *Apc*^Min/+^ littermates. In the current study, we utilized a genetic approach to investigate the effect of CysLT_1_R in the induced azoxymethane/dextran sulfate sodium (AOM/DSS) model of colitis-associated colon cancer. We found that AOM/DSS female mice with a global disruption of the *Cysltr1* gene *(Cysltr1*^−/−^) had a higher relative body weight, a more normal weight/length colon ratio and smaller-sized colonic polyps compared to AOM/DSS wild-type counterparts. The *Cysltr1^−/−^* colonic polyps exhibited low-grade dysplasia, while wild-type polyps had an adenoma-like phenotype. The *Cysltr1^−/−^* colonic polyps exhibited significant decreases in nuclear β-catenin and COX-2 protein expression, while the normal crypts surrounding the polyps exhibited increased Mucin 2 expression. Furthermore, *Cysltr1^−/−^* mice exhibited an overall reduction in inflammation, with a significant decrease in proinflammatory cytokines, polyp 5-LOX expression and infiltration of CD45 leukocytes and F4/80 macrophages. In conclusion, the present genetic approach in an AOM/DSS model further supports an important role for CysLT_1_R in colon tumorigenesis.

## INTRODUCTION

Patients suffering from inflammatory bowel disease (IBD) have an approximately 2- to 5-fold increased risk of developing colorectal cancer compared to age-matched healthy individuals. IBD involves chronic inflammation of the intestinal tract and includes ulcerative colitis (UC) and Crohn's disease (CD) [1]. The risk factors of developing colorectal cancer in IBD patients include a family history of colorectal cancer, early age of onset of colitis and the concomitant presence of primary sclerosing cholangitis. Other established risk factors are the duration, extent and severity of the inflammation [2]. In accordance with the link between chronic intestinal inflammation and the development of colorectal cancer, studies have shown that the treatment of IBD patients with anti-inflammatory amino salicylates reduces the risk of developing colorectal cancer [3]. Additionally, in the azoxymethane/dextran sodium sulfate model of colitis-associated colorectal cancer (CAC), 5-amino salicylic acid has been shown to reduce the number of dysplastic lesions [4].

Colon cancer development has been shown to be promoted by the irregular metabolism of arachidonic acid, an ω-6 family of polyunsaturated essential fatty acids, which ultimately leads to the generation of the proinflammatory lipid mediators prostaglandins and leukotrienes via cyclooxygenase (COX) isozymes 1 and 2 and 5-lipoxygenase (5-LOX), respectively [5]. The leukotrienes (LTs) are generated by 5-LOX, and LTC_4_, LTD_4_ and LTE_4_, are denoted as cysteinyl leukotrienes (CysLTs) due to their cysteine amino acid residue [6]. The involvement of 5-LOX in the chronic inflammatory condition of IBD is evident from the findings of a 3–7-fold increase in 5-LOX expression in the colonic biopsies of patients with active IBD compared to controls and elevated urine levels of LTE_4_ in patients with active UC and CD [7]. In addition to the increased levels of PGE_2_ in the inflamed colonic tissues, elevated levels of leukotrienes (LTB_4_ and CysLTs) could also be detected in a guinea pig model of IBD [8]. In a chemically induced rat model of IBD, the administration of a dual LTC_4_ and LTD_4_ antagonist reduced the colonic damage and inflammation, as evidenced by the reduced levels of LTC_4_ [9].

CysLTs mediate their effects through G-protein coupled receptors (GPCRs), chiefly CysLT_1_R and CysLT_2_R [10]. Increased CysLT_1_R expression has been observed in several human solid tumors, including colorectal cancer [11–16]. We have shown that the up-regulated CysLT_1_R tumor expression in breast and colorectal cancer patients predicts a negative prognosis [11, 16], whereas concurrent low CysLT_1_R and high CysLT_2_R expression in colonic tumors convey a favorable prognosis [17]. We have also shown that the LTD_4_-induced CysLT_1_R signaling is important in promoting colorectal tumorigenesis by increasing the expression of proteins associated with cell survival (COX-2 and Cyclin D1) and increased proliferation and migration (β-catenin) in xenograft colon cancer and colon cancer cell lines [18–20]. In a mouse xenograft study, we demonstrated that the CysLT_1_R antagonist treatment reduces the colon cancer tumor growth by impairing angiogenesis, inducing apoptosis and inhibiting proliferation [21]. In the Apc^Min/+^ mouse model, the loss of function of the tumor suppressor gene *Adenomatous polyposis coli* (*Apc*) is an early event in tumorigenesis, which is consistent with autosomal dominant inherited colorectal cancer (familial adenomatous polyposis; FAP) and sporadic colorectal cancer [22, 23]. Additionally, in an *Apc*^Min/+^ mouse model of sporadic CRC, female double-mutant *Apc*^Min/+^/*Cysltr1*^−/−^ mice developed fewer intestinal polyps compared to their *Apc*^Min/+^ littermates [24]. However, one of the disadvantages of Apc^Min/+^ mice is that they are predisposed to multiple neoplasms (Min) that occasionally progress to adenocarcinomas in the small intestine, although very few neoplastic lesions develop in the colon [25].

Therefore, to study the impact of CysLT_1_R in colitis-associated colorectal cancer, we employed the azoxymethane/dextran sodium sulfate (AOM/DSS) model in mice with disrupted expression of CysLT_1_R. This model is a two-step model where the tumor development in the colon is initiated with the carcinogen azoxymethane and then promoted by the administration of the irritant dextran sodium sulfate [26].

## RESULTS

### AOM/DSS treatment induced colitis in *Cysltr1*^−/−^ female mice and their wild-type counterpart

**Figure 1:**
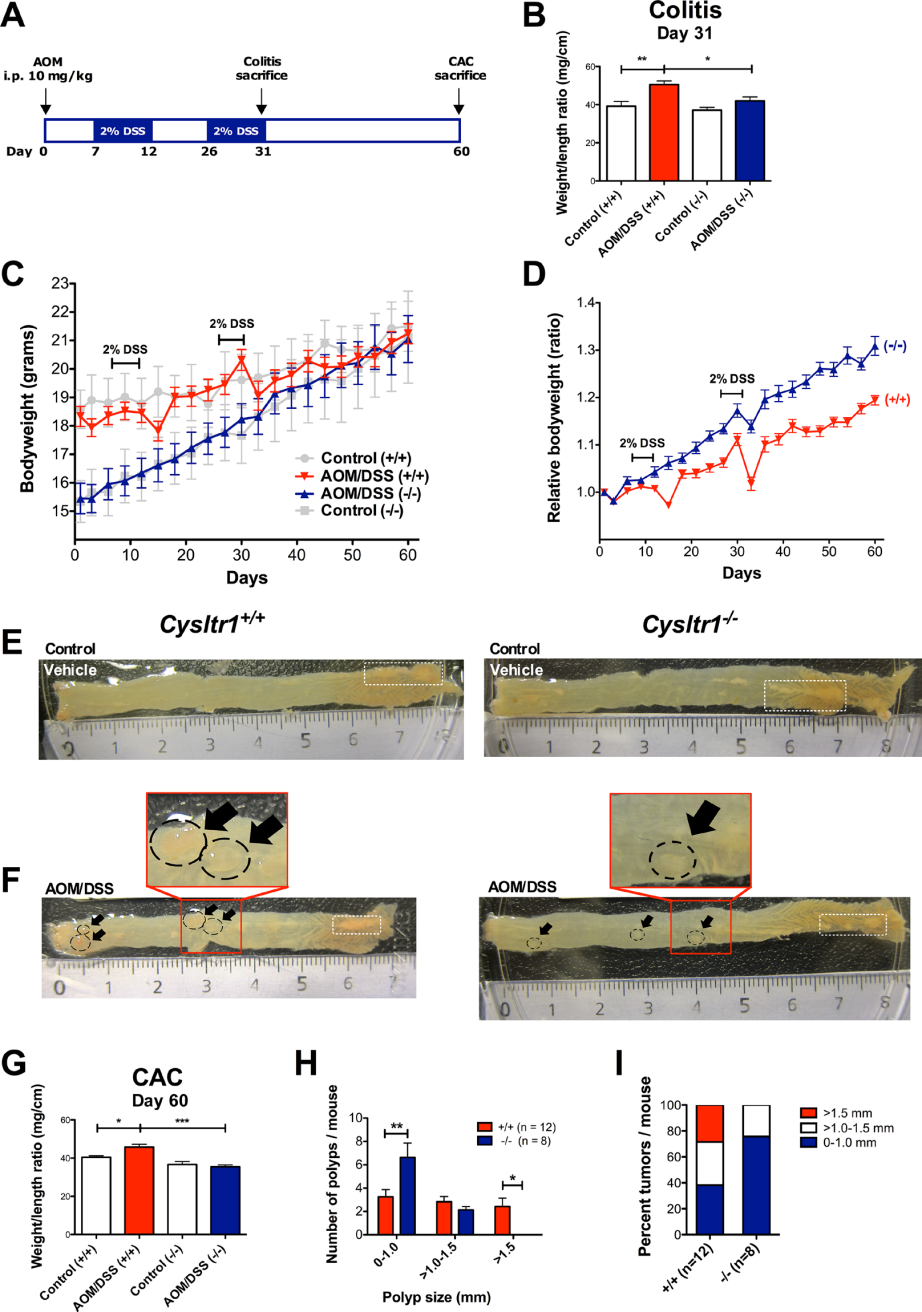
Effect of CysLT_1_R expression on AOM/DSS-induced colitis-associated colon tumorigenesis (**A**) Schematic overview of the AOM/DSS-induced colitis-associated colon cancer model. (**B**) Colon weight/length ratio assessed at colitis end-point (day 31) for untreated vehicle control and AOM/DSS-treated mice (*n* = 5–7 for each genotype). (**C**) Actual mouse body weight (grams) for AOM/DSS and vehicle control female wild type and *Cysltr1*^−/−^ mice. (**D**) Relative body weights of female mice of indicated *Cysltr1* genotypes. For wild-type (+/+) females, 12 mice were euthanized at the late CAC end-point (day 60). For *Cysltr1*^−/−^ (−/−) females, eight mice were euthanized at day 60. Colon weight/length ratio assessed at day 31 for untreated control and AOM/DSS-treated mice (*n* = 5–7 for each genotype). Representative images of (**E**) vehicle control colons from wild type and *Cysltr1*^−/−^ females at day 60 and (**F**) AOM/DSS-treated colons from wild-type and *Cysltr1*^−/−^ mice at CAC end-point. (Polyps are indicated by arrows and fat appendages are indicated by white dotted boxes). (**G**) Colon weight/length ratio assessed at the late end-point (day 60) for vehicle control (*n* = 7–8), AOM/DSS-treated mice (*n* = 8 for wild-type and *n* = 12 for *Cysltr1*^−/−^ mice). (**H**) Polyp size and number in the colon were evaluated for AOM/DSS *Cysltr1*^−/−^ mice and their wild-type counterparts. (**I**) Percentage of polyps per size group for each genotype. Quantitative data are shown as the mean ± SEM. *P < 0.05, **P < 0.01, ***P < 0.001 by unpaired *t-test* and two-way ANOVA for polyp size distribution analysis.

To investigate the role of *Cysltr1* in colitis-associated colorectal cancer, wild-type and CysLT_1_R mutant mice (*Cysltr1*^−/−^) were exposed to AOM and DSS as depicted in Figure 1A. The clinical severity of the colitis was determined by measuring the body weight every third day throughout the experiment (Figure 1C) and calculating the colon weight/length ratio for early (colitis, Figure 1B) and late (CAC, Figure 1G) end-points. The AOM/DSS animals, irrespective of their *Cysltr1* genotype, showed a clear drop in relative body weight after the second DSS cycle, with the *Cysltr1*^−/−^ mice being less affected by the AOM/DSS treatment. The AOM/DSS *Cysltr1*^−/−^ mice euthanized at the early (colitis) and late (CAC) end-points had a significantly decreased colon weight/length ratio compared to their wild-type counterparts (Figure 1B and 1G). This was mainly due to the more normal-sized colons in *Cysltr1*^−/−^ mice (Figure 1E and 1F). Collectively, these results are indicative of induced colitis in the AOM/DSS-treated animals and correspond well to the clinicopathologic signs of chronic ulcerative colitis in mice, as described by Inoue *et al*. [27].

### Smaller colonic polyps were observed in AOM/DSS *Cysltr1*^−/−^ mice

Mice treated with vehicle control did not display any colonic polyps and no increase in inflammatory infiltrate and edema in the submucosa (Figure 1A). No spontaneous polyps were observed in vehicle control mice (Figure 1E). When comparing the mean numbers of polyps for the AOM/DSS *Cysltr1*^−/−^ mice and their wild-type counterparts (*Cysltr1*^+/+^), no significant difference was observed, although the *Cysltr1*^−/−^ mice clearly exhibited polyps of a smaller size (Figure 1H). The AOM/DSS *Cysltr1*^−/−^ mice mainly exhibited colonic polyps of <1 mm in diameter with no polyps exceeding 1.5 mm, while the wild-type (*Cysltr1*^+/+^) counterparts exhibited all sizes of polyps, including polyps exceeding 1.5 mm in size (Figure 1H and 1I).

**Figure 2:**
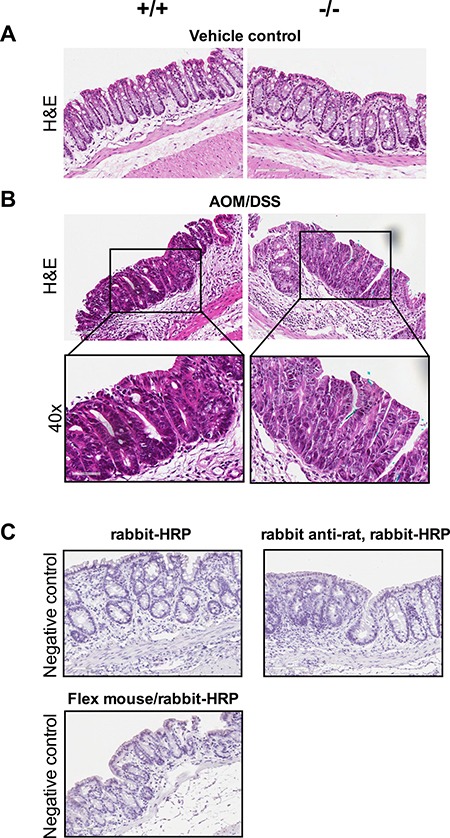
Dysplastic lesions/ACFs developed in AOM/DSS mice independent of *Cysltr1* status The colons of vehicle control and AOM/DSS mice with different *Cysltr1* genotype were processed for immunostaining. (**A**) Representative images (20×) of hematoxylin and eosin staining of vehicle control colons with no observed polyps. (**B**) Representative images (20× and 40×) of AOM/DSS female mice with different *Cysltr1* genotypes with dysplastic lesions/ACFs stained for hematoxylin and eosin (*n* = 3–6 per genotype). (**C**) Negative controls (IgG controls) for all the secondary antibodies used, rabbit-horseradish peroxidase (HRP), rabbit anti-rat with rabbit-HRP, and combined Flex mouse/rabbit-HRP.

Figure 2A showing vehicle control normal areas stained with hematoxylin and eosin (H&E) *Cysltr1*^−/−^ mice and their wild-type counterparts (*Cysltr1*^+/+^), The AOM/DSS treatment induced dysplastic lesions/aberrant crypt foci (ACF) in the mouse colons, and H&E staining revealed that the wild-type polyps were serrated adenomatous structures, while the *Cysltr1*^−/−^ polyps were low-grade dysplastic in nature (Figure 2B). Shortening of the colon length in DSS mice is one of the biological markers of severity of colonic inflammation [27] and our results clearly show that the *Cysltr1*^−/−^ mice have a less severe colitis with a more normal-like colon length (with reduced colonic inflammation and edema, see Figure 2) and a lower grade of dysplasia compared to the wild-type mice. Figure 2C shows negative IgG control stainings.

### Decreased expression of nuclear β-catenin and COX-2 in tumors of AOM/DSS *Cysltr1*^−/−^ mice

**Figure 3:**
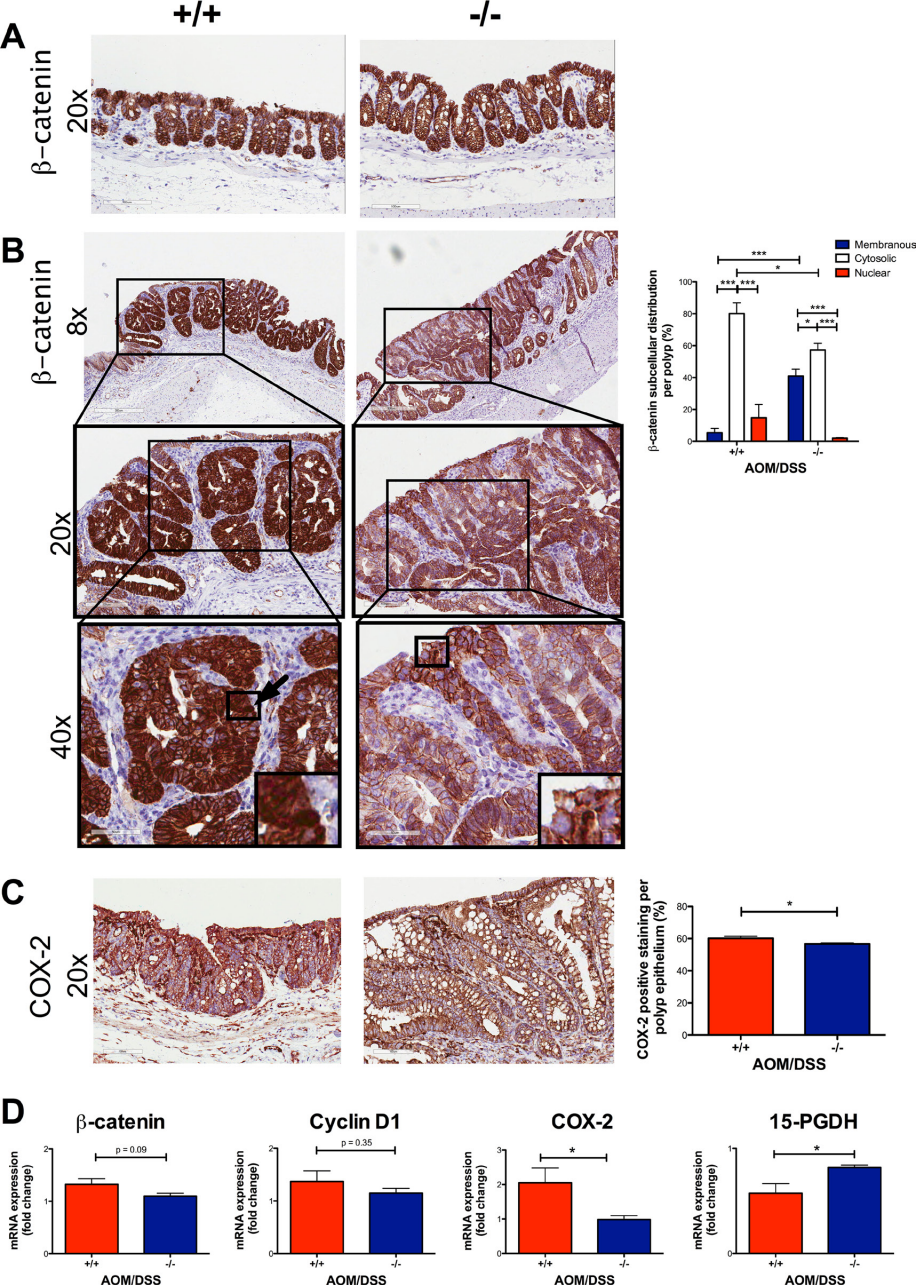
Decreased expression of nuclear β-catenin and COX-2 in AOM/DSS *Cysltr1*^−/−^ mice The colons of AOM/DSS female mice with different *Cysltr1* genotypes (*n* = 3–6 per genotype) were processed for immunostaining. Representative images of (**A**) non-polyp area stained with β-catenin (at 20×), (**B**) polyp area stained with β-catenin (at 8×, 20×, 40×), (**C**) COX-2 and their corresponding bar diagrams showing the percentages of (B) subcellular localization of epithelial β-catenin and (C) COX-2 positive epithelial cells within polyps. (**D**) Relative colon mRNA expression was determined with qPCR for female mice with indicated *Cysltr1* genotypes (*n* = 5–7) and normalized against *Gapdh* and untreated wild-type female for *β-catenin*, *cox-2*, *15-PGDH* and *cyclin D1*. Quantitative data are shown as the mean ± SEM. *P < 0.05, **P < 0.01, ***P < 0.001 by one-way or two-way ANOVA for immunohistochemistry and by *t*-test or Mann-Whitney test for mRNA data.

The nuclear localization of β-catenin is believed to be required for the CRC progression and is found in late adenomas and carcinomas [28]. We, therefore, investigated the effect of the global *Cysltr1* gene disruption on tumors by antibody immunostaining for β-catenin. No difference was observed in β-catenin localization of non-polyp areas in AOM/DSS mice (Figure 3A). In polyps, a difference in the tumor subcellular localization of the expressed β-catenin could be observed. The *Cysltr1*^−/−^ mice had increased membranous and decreased nuclear β-catenin expression compared to the wild-type mice (Figure 3B). However, when analyzing the *Cysltr1*^−/−^ colons with identified tumors, the total *ctnnb1* (β-catenin) mRNA expression was not significantly reduced compared to that of the wild-type mice (Figure 3D). Therefore, the smaller polyps observed in the *Cysltr1*^−/−^ mice could be due to the decreased nuclear accumulation of β-catenin.

When β-catenin translocates to the nucleus, it can act as a transcription factor, and one of its target genes is cyclooxygenase-2 (COX-2) [29]. Therefore, we investigated if there was any change in the COX-2 expression in the tumor epithelium. We stained and assessed the COX-2 expression in the tumors. The colonic tumors of the AOM/DSS *Cysltr1*^−/−^ mice had significantly decreased COX-2 protein expression (*P* < 0.05) compared to the wild-type mice (Figure 3C). The *Cysltr1*^−/−^ colons also displayed a decrease in the *ptgs2* (COX-2) mRNA expression (Figure 3D). No difference was seen in *cyclin D1*, an additional β-catenin target gene, but an increase was seen in the enzyme responsible for prostaglandin E_2_ catabolism, 15-hydroxyprostaglandin dehydrogenase (15-PGDH) (Figure 3D) [30].

### Increased expression of Muc-2 in crypts of *Cysltr1*^−/−^ mice

**Figure 4:**
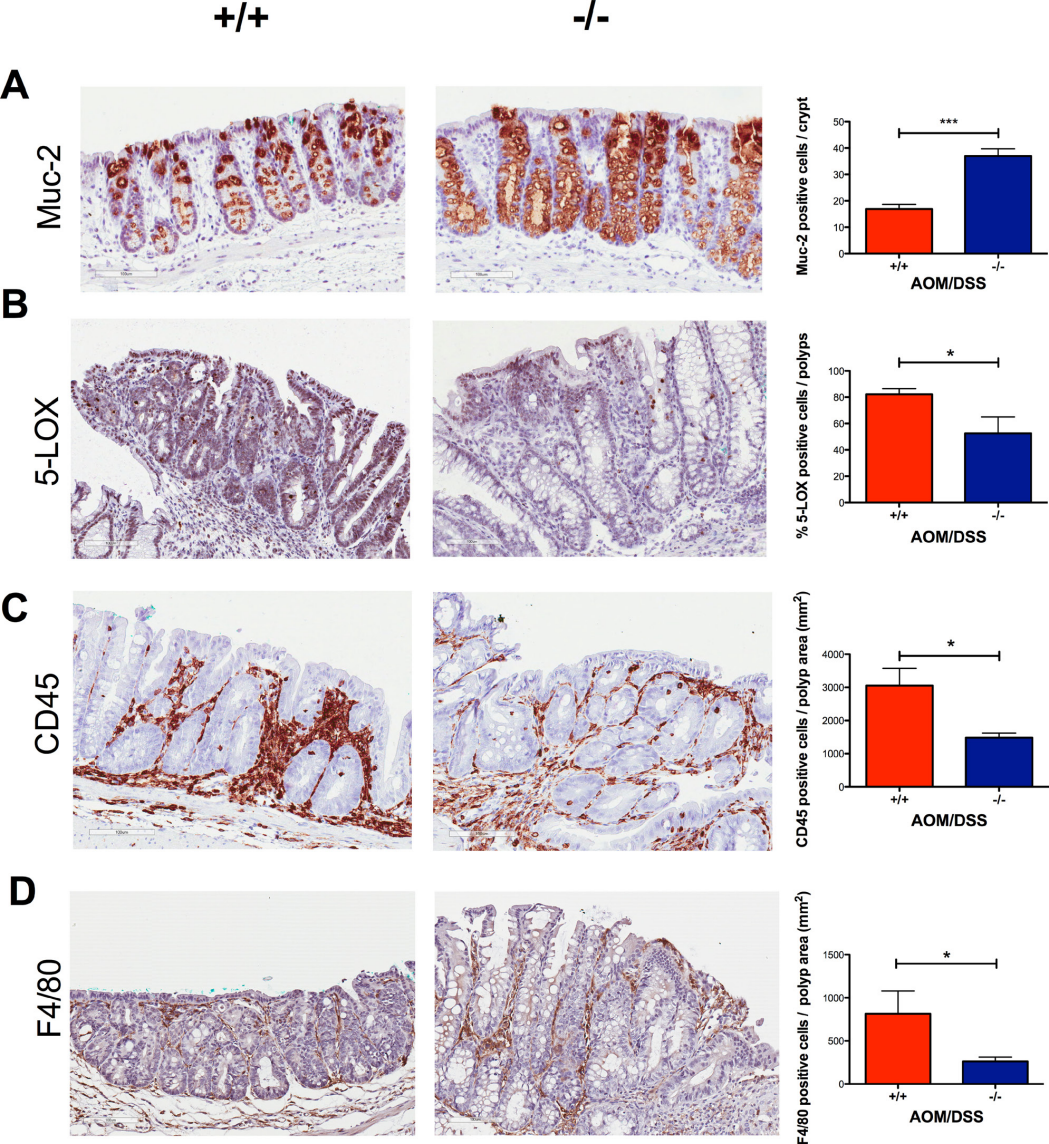
Decreased inflammation in AOM/DSS *Cysltr1*^−/−^ colons with less infiltration of immune cells and increased expression of Mucin 2 Representative images (20×) of colonic crypts stained with (**A**) mucin 2, and colonic polyps stained with (**B**) 5-LOX, (**C**) CD45, and (**D**) F4/80 with their corresponding bar diagrams showing (A) mucin 2 positive cells per normal crypt, (B) the percentages of 5-LOX positive cells, (C) CD45 positive cells per polyp area (mm^2^) and (D) F4/80 positive cells per polyp area (mm^2^). Quantitative data are shown as the mean ± SEM, where *n* = 2–6 per genotype. *P < 0.05, ***P < 0.001 by *t*-test or Mann-Whitney test.

Mucin 2 (Muc-2) is the most prominent secreted gastrointestinal mucin and a differentiation marker for goblet cells. The reduced number of Muc-2 producing goblet cells is characteristic of aberrant crypt foci (ACF), pre-neoplastic lesions that occur in both humans and rodents [31–33]. We observed an increased expression of Muc-2 (*P* < 0.05) in the crypts surrounding the polyps in *Cysltr1*^−/−^ mice compared to those of their wild-type counterparts (Figure 4A).

### Reduced expression of 5-LOX and decreased immune cell infiltration in tumors of AOM/DSS *Cysltr1*^−/−^ mice

**Figure 5:**
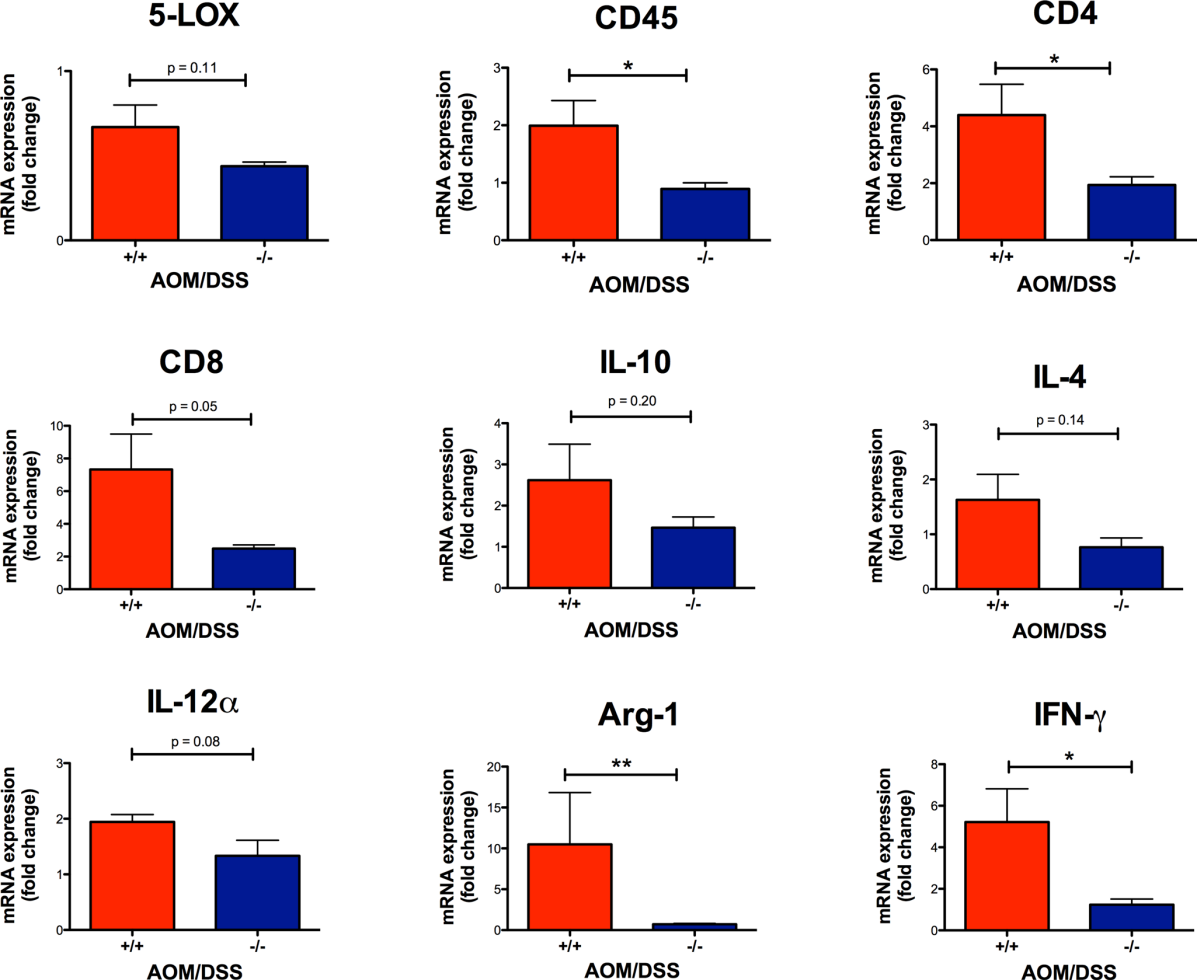
Effect of CysLT_1_R on mRNA levels of AOM/DSS mouse colons Relative colon mRNA expression was determined with qPCR for AOM/DSS female mice with indicated *Cysltr1* genotypes (*n* = 5–7) and normalized against *Gapdh* and untreated wild-type female for *5-LOX, CD45, CD4, CD8, IL-10, IL-4, IL-12α, Arg-1 and IFN-γ*. Quantitative data are shown as the mean ± SEM. *P < 0.05, **P < 0.01 by *t*-test or Mann-Whitney test.

To investigate the effect of CysLT_1_R on the inflammatory status in AOM/DSS mice, we performed immunostaining and assessed the expression of the enzyme responsible for CysLT production, 5-LOX, in tumors. The colonic tumors of the AOM/DSS *Cysltr1*^−/−^ mice had significantly decreased 5-LOX protein expression (*P* < 0.05) compared to their wild-type counterparts (Figure 4B). However, the *Cysltr1*^−/−^ colons with tumors did not display a significant decrease in the *alox5* (5-LOX) mRNA expression compared to those of the wild-type mice (Figure 5).

Immune cells are known to infiltrate tumors [34], and the CysLT_1_R is known to be expressed on specific leukocytes, such as monocytes and macrophages [35]. Therefore, we decided to investigate the infiltrating immune cells in wild-type and *Cysltr1*^−/−^ tumors. The AOM/DSS *Cysltr1*^−/−^ mice had fewer tumor-infiltrating CD45-positive leukocytes compared to their wild-type counterparts (Figure 4C), which is further supported by the significantly decreased *ptprc* (CD45) mRNA expression of colons with identified tumors (Figure 5). Moreover, the *Cysltr1*^−/−^ polyps had a significantly decreased tumor infiltration of F4/80-positive macrophages compared to those of the wild-type mice (Figure 4D). Upon further examination of the different immune cell subgroups, the *Cysltr1*^−/−^ colons displayed a significant decreased *CD4* and *Arg-1* mRNA expression, which is indicative of mainly CD4^+^ T cells and type-2 macrophages, respectively, with a modest decrease in *CD8* mRNA levels that was not quite significant (Figure 5).

### Inflammatory cytokines in the colon and serum

**Figure 6:**
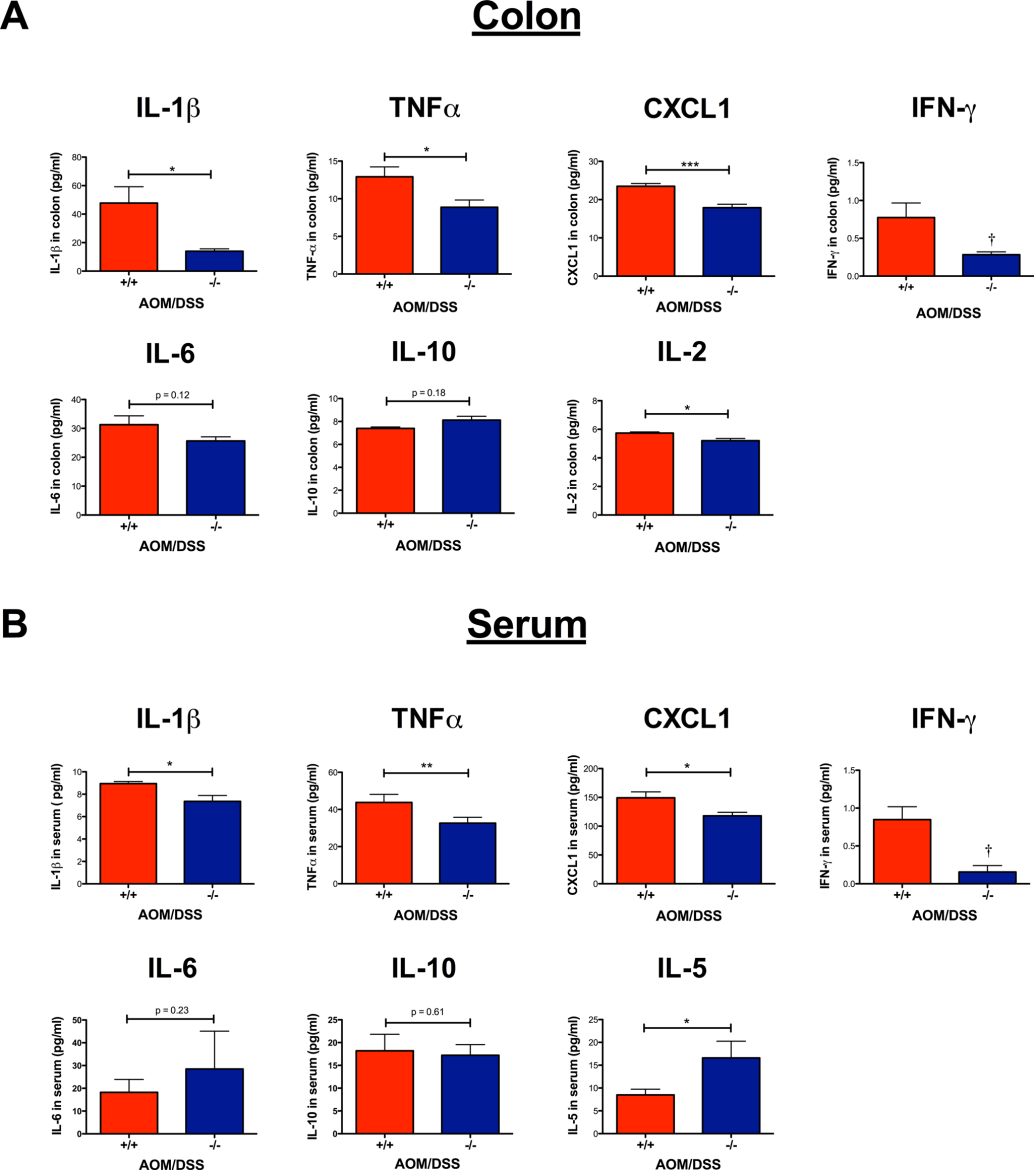
Effect of CysLT_1_R on proinflammatory mediators detected in AOM/DSS colon and serum Cytokines were quantified from the AOM/DSS serum (*n* = 3–7) and colon samples (*n* = 2–7) using a multiplex sandwich immunoassay format. (**A**) Inflammatory mediators (IL-1β, TNFα, CXCL1, IFN-γ, IL-6, IL-10 and IL-2) quantified from colon lysates. (**B**) Inflammatory mediators (IL-1β, TNFα, CXCL1, IFN-γ, IL-6, IL-10 and IL-5) quantified from serum. Quantitative data are shown as the mean ± SEM. *P < 0.05, **P < 0.01, ***P < 0.001 by *t*-test or Mann-Whitney test. Values below the detection level were labeled as (†).

The inflammatory response is known to either promote or inhibit tumorigenesis. We, therefore, investigated how the loss of functional CysLT_1_R affected the proinflammatory cytokines both locally in the colon tissue and systemically in the serum. In *Cysltr1*^−/−^ colon and serum samples, a significant decrease was seen in IL-1β, TNFα and CXCL1 compared to those from the wild-type mice (Figure 6A and 6B). For the wild-type mice, an increased level of IFN-γ was seen in both the colon (mean of 0.77 pg/ml) and serum (mean of 0.85 pg/ml), while all *Cysltr1*^−/−^ IFN-γ levels fell below the detection range (< 0.22 pg/ml) (Figure 6A and 6B). This seemingly low IFN-γ protein expression in the *Cysltr1*^−/−^ mice was further supported by the significant decrease in *IFN-γ* mRNA expression found in the *Cysltr1*^−/−^ colons compared with those of the wild-type mice (Figure 5). No significant difference was detected in colon *IL-10* mRNA levels (Figure 5) and in colon and serum IL-6 or IL-10 levels for mice, independent of their CysLT_1_R status (Figure 6A and 6B). Interestingly, for the *Cysltr1*^−/−^ mice, only the colon samples exhibited a decrease in IL-2 (Figure 6A), while only the serum samples exhibited an increase in IL-5 compared to the wild-type colons (Figure 6B). The levels of IL-4 and IL-12 fell below the detection range for both colon and serum samples, and serum IL-2 and colon IL-5 levels also fell below the detection range for all mouse samples.

## DISCUSSION

Studies have shown that the administration of DSS and the induction of inflammation in mice that are genetically susceptible to intestinal cancer promote the formation of dysplastic lesions in the colon [36, 37]. Therefore, previous findings from our group regarding CysLT_1_R signaling indicates that it could increase the susceptibility to colitis-associated colorectal cancer [21]. We have previously demonstrated that the CysLT_1_R has a tumor-promoting role in the Apc^Min/+^ mouse model of intestinal tumorigenesis [24]. The mechanism of tumor progression in colitis-associated colon cancer (CAC) differs, however, from that of hereditary and sporadic colorectal cancer, exemplified by the early loss of *APC* in sporadic pathogenesis, which occurs later in CAC [23].

In the current study, we investigated the effect of the CysLT_1_R by a genetic approach. In an AOM/DSS-induced colitis-associated colon cancer model, we found that female mice with global *Cysltr1* gene disruption had less severe disease activity compared with wild-type mice. This indicates that *Cysltr1*^−/−^ mice have a more favorable outcome, particularly due to their smaller colonic polyps, which are more dysplastic in morphology compared to the adenomas in the wild-type mice.

We have previously demonstrated that the LTD_4_-mediated CysLT_1_R activation induces proliferation in the intestinal epithelial cells and colon cancer cells. This proliferation can be inhibited *in vivo* by CysLT_1_R antagonist treatment [21]. Accordingly, the LTD_4_-stimulation of CysLT_1_R in colon cancer cells induces β-catenin nuclear accumulation and transcriptional activity in colon cancer cells with subsequently increased proliferation, migration and invasion with induction of epithelial-mesenchymal transition [19, 38]. We did not observe any difference in β-catenin localization in non-polyp areas of AOM/DSS wild type and *Cysltr1*^−/−^ colons. AOM/DSS *Cysltr1*^−/−^ mice exhibited an increased membranous and decreased nuclear β-catenin expression of the polyp epithelium, with a tendency for decreased total *ctnnb1* (β-catenin) mRNA levels, and with decreased COX-2 levels. This implies less translocation of β-catenin to the nucleus, which in turn would suggest a lower activation of the β-catenin target genes that can promote tumor proliferation and migration.

One important target gene of β-catenin is COX-2 [29], and the increased expression of COX-2 is necessary for tumors to progress from adenomas to carcinomas [34]. Additionally, in the AOM/DSS mice, increased expression of COX-2 and 5-LOX has been detected in colonic tumors compared with in the surrounding normal mucosa [39]. In patients, increased 5-LOX levels were detected in colon cancer samples compared with in the normal surrounding mucosa, which was correlated with increased CysLT_1_R expression [11]. In our AOM/DSS mouse model, the colonic polyps of *Cysltr1*^−/−^ mice displayed decreased protein and mRNA expression of *COX-2* and *5-LOX*, with increased mRNA levels of the tumor suppressor *15-PGDH* [30], which together imply a better outcome, even though no difference was seen in colonic CysLT_2_ receptor expression between vehicle control and AOM/DSS treated wild type or *Cysltr1*^−/−^ mice (Supplementary Figure 1). Others have shown using *Cox-2* knockout mice with DSS-induced colitis that the myeloid and endothelial cell expression of COX-2 has a protective role against colon inflammation [40, 41]. Furthermore, the COX-2 expression and prostanoids derived from the cyclooxygenase pathway were shown to not be essential in colitis-associated cancer in AOM/DSS-treated *Cox-2* knockout mice [42]. However, because the β-catenin activation is important in the early stages of colon tumorigenesis [34], we consider the decrease of COX-2 in *Cysltr1*^−/−^ polyps to be more favorable.

The important protective role of Mucin 2 (Muc-2) in colorectal cancer has been demonstrated in Muc2-deficient mice, which develop spontaneous intestinal and rectal tumors that eventually progress to carcinomas [43]. In our study, *Cysltr1*^−/−^ mice displayed significantly increased expression of Muc-2 from normal crypts surrounding the polyps. This is interesting, because the loss of Muc-2 expression contributes to the colorectal cancer progression, and this loss could be facilitated by Wnt/β-catenin signaling [44].

In colorectal cancer patients, the frequency and subtype of the tumor-infiltrating lymphocytes have been shown to predict prognosis [45]. Additionally, DSS treatment has been shown to induce acute inflammation in the colon, with increased mucosal permeability, damage and leukocyte infiltration [46]. We, therefore, investigated the tumor infiltration of leukocytes and the expression of different inflammatory mediators in AOM/DSS mice. We observed a decreased polyp infiltration of immune cells (CD45 leukocytes and F4/80 macrophages) in the *Cysltr1*^−/−^ polyps. Since a decrease was also seen in colon mRNA levels of *CD4* and *Arg-1*, it is plausible that the decreased polyp infiltrate also consisted of CD4^+^ T cells and type-2 macrophages [47]. Previous patient data from our group have shown that tumor associated macrophages (type-2 phenotype) heavily infiltrate tumors and are associated with a poorer prognosis for colon cancer patients [48]. This would suggest a favorable outcome in *Cysltr1*^−/−^ mice, with less polyp infiltrated macrophages and less colon *Arg-1* levels compared to wild type mice. Additionally, in an Apc^Min+^ model of colon cancer, *Apc*^Min/+^/*Cysltr1*^−/−^ mice tended to have fewer CD3^+^CD4^+^ T cells infiltrating polyps [24]. The decrease in *CD4* and *Arg-1* in *Cysltr1*^−/−^ mice could be explained by the finding that CD4^+^ Th2 cells can facilitate the expression of arginase-1 in murine macrophages, and arginase itself can promote tumor growth and the down-regulation of tumor cytotoxicity [49]. High IL-10 levels have shown to be secreted from colon cancer cells to promote M2 macrophage differentiation [48]. We did, however, not observe any difference in mRNA or protein expression levels of IL-10, between *Cysltr1*^−/−^ and wild type mice, and observed higher mRNA and protein expression levels of IFN-γ, in wild-type mice. This would indicate a decrease of both type-1 and type-2 macrophages in *Cysltr1*^−/−^ mice compared to wild type [50]. Furthermore, IL-5, which can be produced by mast cells and eosinophils, has been shown to promote eosinophil infiltration into tumors and suppress tumor metastasis [51]. We have previously shown that an increased mast cell density has been associated with a better prognosis in colorectal cancer patients, and an increased mast cell number was seen in tumors of AOM/DSS *Cysltr1*^−/−^ mice [52]. Taken together, the increased mast cell number and increased IL-5 serum levels indicate a more favorable outcome for *Cysltr1*^−/−^ mice.

At the initial stage of CAC, most cytokines responsible for tumorigenesis are produced by lamina propria macrophages and dendritic cells [34]. We observed an increased expression of the neutrophil chemoattractant CXCL1 in wild type mice, whose expression has been shown to increase in adenomas and adenocarcinomas, indicating a more advanced dysplastic lesions/ACFs, which is supported by our immunohistochemistry data. Wild type mice also exhibited an increase in the proinflammatory cytokines TNFα and IL-1β, which together can further up-regulate the CXCL1 levels [50]. TNFα is known to have many effects, and it has be involved in the nuclear accumulation of β-catenin and increased COX-2 expression in AOM/DSS mice [53] which was also evident in the current AOM/DSS wild-type mice. Additionally, IL-1β blockade significantly decreased tumor development in another AOM/DSS mouse model [54], which might explain the less dysplastic lesions found in *Cysltr1*^−/−^ polyps. Furthermore, *in vitro* studies with the addition of type-2 macrophage medium, TNFα or LTD_4_ to colon cancer cells also induced nuclear accumulation of β-catenin and increased invasiveness [38]. This might indicate that the type-2 macrophages secrete TNFα and LTD_4_, which can promote more dysplastic polyps partly via CysLT_1_R-signaling.

In conclusion, the present genetic approach in a colitis-associated colon cancer model further supports an important role for CysLT_1_R signaling in colon tumorigenesis, driving immune mediators to promote immune cell infiltration in tumors.

## MATERIALS AND METHODS

### Animals

*Cysltr1* gene disrupted (*Cysltr1*^−/−^) mice on a C57BL/6N background were kindly provided by Prof. Frank Austen (Harvard Medical School, Brigham and Women's Hospital, Boston, MA, USA) [53]. A breeding colony was established and maintained at the Lund University Animal Facility, Malmö. All mice were housed in individually ventilated cages, in facilities equipped with 12-h light and dark cycles. All offspring were genotyped for the *Cysltr1* allele in genomic DNA isolated from ear biopsies using *polymerase chain reaction* (PCR), as previously described [24]. All animal experiments were conducted according to D.Nr. M262–12 and were approved by the Regional Ethical Committee for Animal Research at Lund University, Sweden.

### Experimental procedure

Female 6- to 8-week-old wild type and *Cysltr1*^−/−^ mice were given a single intraperitoneal injection of 10 mg/kg azoxymethane (AOM) (Sigma-Aldrich Corporation, St. Louis, MO, USA) in 0.9% NaCl, followed one week later by two 5-day cycles of 2% dextran sodium sulfate (DSS) (MP Biomedicals, Santa Ana, CA, USA) in drinking water *ad libitum*, with an intermediate recovery period of two weeks (Figure 1A). The control mice received a vehicle (0.9% NaCl) intraperitoneal injection and were not given the DSS treatment. The body weight of the mice was recorded every third day. The mice were euthanized by CO_2_ asphyxiation either 31 days (early colitis end-point) or 60 days (late CAC end-point) after the initial AOM/vehicle injection. The complete colon was excised (from anus until cecum) and most of the fat appendages removed without damaging the colon, rinsed with ice-cold phosphate-buffered saline, and opened longitudinally, and the weight and length were recorded. Approximately half of the experimental animals per group (genotype) had their colon fixed flat in 10% formalin overnight and stored in 70% ethanol. The size and number of colonic tumors were evaluated using a dissection microscope (20×), and the colon was finally subdivided into smaller pieces (approx. 2 cm) and finally embedded in paraffin. The rest of the animals had their colon snap-frozen in 1- to 2-cm pieces in liquid nitrogen and stored at −80°C for subsequent RNA extraction and protein quantification.

### Immunohistochemistry

Sectioned (5 μm) paraffin-embedded colonic tissues were immunostained using a Dako automatic slide stainer according to the manufacturer's instructions. The sections were stained with rabbit anti-human COX-2 polyclonal antibody (dilution 1:200; Abcam, Cambridge Science Park, Cambridge, UK), rabbit anti-human/mouse 5-LOX polyclonal antibody (dilution 1:100; Cayman Chemical, Ann Arbor, MI, USA) and anti-human/mouse β-catenin (dilution 1:1000; BD Biosciences, Franklin Lakes, NJ, USA), both anti-human/mouse CD45 polyclonal antibody (dilution 1:100) and anti-human/mouse Mucin 2 polyclonal antibody (dilution 1:400) from Santa Cruz Biotechnology, Santa Cruz, CA, USA), and rat anti-mouse Anti-F4/80 monoclonal antibody (dilution 1:100; Abcam, Cambridge Science Park, Cambridge, UK). All stainings were analyzed on digitally scanned slides (ImageScope v11.2.0.780, Aperio Technologies, Inc., Vista, CA, USA) by two independent investigators blinded to the *Cysltr1* status. The expression of 5-LOX within the dysplastic lesions was estimated as the percentage of positive-stained cells per polyp. The expression of COX-2 was evaluated by an image-analyzing program HALO v2.0 (Indica Labs; Albuquerque, NM, USA) software to obtain the percentage of COX-2-positive epithelial tissue within the annotated epithelial polyps and is based on pixel algorithms. Epithelial cells were identified with the aid of the Classifier module, and the COX-2 positive staining was detected using the AreaQuantification algorithm, with a manually adjusted positive-staining threshold. The subcellular expression of β-catenin was assessed within the positive epithelial cells of the polyp. The Muc-2 expression was evaluated as the number of positive cells per crypt, where ten crypts per polyp were assessed. CD45 and F4/80 immunostaining was evaluated as the number of positive cells per polyp area. IgG controls (negative controls) were stained for by using rabbit-HRP, Flex mouse/rabbit-HRP and rabbit anti-rat with subsequent rabbit-HRP staining (all from Dako) (Figure 2C).

### Quantitative RT-PCR

The total RNA was extracted from frozen colon tissue according to the manufacturer's protocol using the RNeasy Plus Mini kit (Qiagen, Hilden, Germany). 1.0 μg RNA was used for the cDNA synthesis using RevertAid H Minus M-MuLV Reverse Transcriptase (Thermo Scientific, Waltham, MA). Quantitative PCR was performed using Maxima probe/ROX qPCR master mix and 0.9 μM TaqMan probes in a 25-μl reaction volume. *The Ta*qMan probes used for *ctnnb1 (β-catenin), ptgs2* (*COX-2)*, *hpgd (15-PGDH)*, *ccnd1 (cyclin D1)*, *alox5* (*5-LOX), ptprc (CD45)*, *CD4, CD8α, IL-12α, arginase-1 (Arg-1), IFN-*γ, *IL-4* and *IL-10* gene expression were Mm00483039_m1, Mm00478374_m1, Mm00515121_m1, Mm01182747_m1, Mm00432359_m1, Mm01293577_m1, Mm00442754_m1, Mm01182108_m1, Mm00434169_m1, Mm00475988_m1, Mm01168134_m1, Mm00445259_m1 and Mm01288386_m1, respectively (Applied Biosystem, Life Technologies, Waltham, MA). Amplification was performed in a Mx3005P thermocycler (Agilent Technologies, Inc., Santa Clara, CA, USA) using MxPro software (Invitrogen Corp, Carlsbad, CA, USA), and a comparative 2-(ΔΔCt) method was used to analyze the data. All data were normalized against the housekeeping gene *Gapdh* (glyceraldehyde-3-phosphate dehydrogenase; Mm99999915_g1) and vehicle control wild type mice.

### Cytokine immunoassay

Blood was collected by cardiac puncture at the time of animal euthanasia, and the non-selective COX inhibitor indomethacin (Sigma–Aldrich, St Louis, MO) was immediately added to the blood samples (at a final concentration of 10 μg/ml), which were allowed to clot for 30 min on ice. Serum was collected after centrifugation in serum separator tubes (Becton Dickinson, Franklin Lakes, NJ, USA) at 6000 *g* for 2 min. The frozen colon tissue samples with identified polyps (around 100 mg) were cut into small pieces and placed in 600 μl lysis buffer for homogenization with a sonicator (VirTis VirSonic 50). The sonicated samples were then incubated while mixing at 4°C for 20 min and then centrifuged at 20,000 *g* for 15 min at 4°C for supernatant collection.

Ten cytokines were quantified from 4-fold and 2-fold diluted serum or colon samples (run in duplicate or triplicate) using a multiplex sandwich immunoassay format and the electro-chemiluminescence MSD ultrasensitive proinflammatory multiplex kit (Meso-Scale Discovery, Gaithersburg, MD, USA). The MSD multispot array was run according to the manufacturer's protocol. Briefly, 96-well plates pre-coated with capture antibodies for TNFα, IL-1β, IL-12 (total), IL-2, IL-4, IL-5, IL-6, IL-10 and CXCL1 and INF-γ were incubated with serum or colon lysate samples for 2 h. Subsequently, detection antibodies were added, and the plate was incubated for another 2 h. After washing, the plate was read using an MS2400 imager (MSD).

### Statistical analysis

All statistical analyses were performed using GraphPad Prism version 5.0a (GraphPad *Software*, Inc., San Diego, CA, USA). The unpaired *t-test* (Student's *t-test*) or Mann-Whitney test was used to compare two groups, and the one-way or two-way ANOVA was used to compare more than two groups. A difference was considered significant when the *P-value* was < 0.05. The data are presented as the mean ± standard error of the mean (SEM).

## SUPPLEMENTARY MATERIALS FIGURES AND TABLES



## Abbreviations

CAC: colitis-associated colorectal cancer
Cysltr1: Cysteinyl leukotriene receptor 1
IL-1β: interleukin-1β
TNFα: tumor necrosis factor α
INF-γ: interferon γ
CXCL1: chemokine (C-X-C motif) ligand 1
IL-6: interleukin 6
IL-10: interleukin 10
IL-2: interleukin 2
IL-5: interleukin 5
IL-12 (total): interleukin 12 (total)
IL-4: interleukin 4
