# Supplementary material for: Cysteinyl leukotriene receptor 1 facilitates tumorigenesis in a mouse model of colitis-associated colon cancer
Source: Oncotarget. 2017 Mar 30;8(21):34773–86. doi: 10.18632/oncotarget.16718 (PMC5471010; doi:10.18632/oncotarget.16718)
Supplement: Supplementary file 1 [file oncotarget-08-34773-s001.pdf]

Cysteinyl leukotriene receptor 1 facilitates tumorigenesis in a mouse model of colitis-associated colon cancer

Supplementary Materials

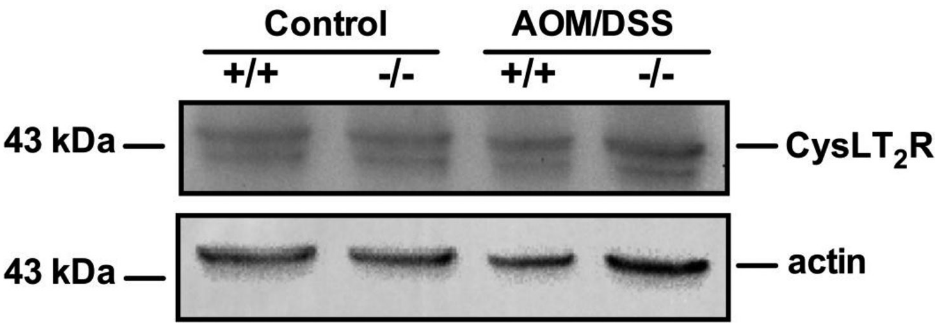

Supplementary Figure 1: Western blot of CysLT$_2$R and actin on colon lysate from vehicle control and AOM/DSS treated wild type (+/+) or *Cysltr1*$^{-/-}$ (–/–) female mice. The lysed colon tissue, as described for the cytokine immunoassay, was subjected to SDS-PAGE and the PVDF membrane was immunoblotted with a CysLT$_2$R (F-15) antibody (1:500) from Santa Cruz, and after stripping, re-incubated with an actin (I-19) antibody (1:3000), also from Santa Cruz, to ensure equal loading.
